# Haemodynamics and Flow Modification Stents for Peripheral Arterial Disease: A Review

**DOI:** 10.1007/s10439-015-1483-4

**Published:** 2015-10-14

**Authors:** Efstratios Kokkalis, Nicolas Aristokleous, J. Graeme Houston

**Affiliations:** Division of Cardiovascular and Diabetes Medicine, Ninewells Hospital and Medical School, University of Dundee, Mail Box 1, Dundee, DD1 9SY United Kingdom

**Keywords:** Peripheral arterial disease, Endovascular, Restenosis, Wall shear stress, Spiral flow, Helical flow, Stent design, Flow modification, Helical stent, Spiral flow stent, Flow optimisation

## Abstract

Endovascular stents are widely used for the treatment of peripheral arterial disease (PAD). However, the development of in-stent restenosis and downstream PAD progression remain a challenge. Stent revascularisation of PAD causes arterial trauma and introduces abnormal haemodynamics, which initiate complicated biological processes detrimental to the arterial wall. The interaction between stent struts and arterial cells in contact, and the blood flow field created in a stented region, are highly affected by stent design. Spiral flow is known as a normal physiologic characteristic of arterial circulation and is believed to prevent the development of flow disturbances. This secondary flow motion is lost in atheromatous disease and its re-introduction after endovascular treatment of PAD has been suggested as a method to induce stabilised and coherent haemodynamics. Stent designs able to generate spiral flow may support endothelial function and therefore increase patency rates. This review is focused on secondary flow phenomena in arteries and the development of flow modification stent technologies for the treatment of PAD.

## Introduction

Peripheral arterial disease (PAD) is recognised as an independent risk factor for both myocardial and cerebrovascular events which are the leading causes of mortality worldwide. PAD can seriously impair the quality of patients’ lives and can lead to leg ulceration, gangrene and eventually amputation. It is the result of any disease that can lead to impaired blood supply, of which atherosclerosis is the main aetiology.^1^,^62^,^80^ Current clinical practice has shown an increased use of stents as a treatment for PAD. The global PAD stent market is expected to rise from $2.2bn in 2012 to $3.6bn in 2019.^27^

There are three main arterial sites where stents are used for PAD; iliac (pelvis), femoropopliteal (thigh) and infrapopliteal (calf). The iliac and femoropopliteal are the sites most commonly stented. Currently, the majority of peripheral vascular stents used at these sites are the bare metal stents most of which are made from nitinol (nickel titanium shape memory alloy). The use of covered stents is the second most common option, followed by drug-eluting and bioabsorbable stents which are expected to be used more in the future.^27^ Poor patency rates of stents for PAD remain a challenge and vary in relation to severity of the disease and its location. In general, proximal infrainguinal stented locations provide better patency rates than distal infrainguinal locations. Stents for PAD are associated with increased rates of fracture because of arterial compression, torsion, bending and contraction in lower extremities.^1^,^18^,^49^,^62^

Stent implantation causes arterial structural injury, introduces a foreign body and affects the local haemodynamics. The result is the activation of four biological responses; thrombosis, neo-intimal hyperplasia (NIH), inflammation and re-endothelialisation. These responses can cause re-narrowing of the stented region, which is known as in-stent restenosis, or activate the development of PAD downstream from the treated region.^59^ Furthermore in relation to local haemodynamics, stents in infrainguinal arteries do not support the re-introduction and propagation of the physiologic rotational secondary flow motions which may affect their long term clinical results.

The aim of this paper is to review the arterial haemodynamics and particularly the nature of secondary flow motions in the arterial system. In addition, to present the effects of stent design in local haemodynamics and review the current state of flow modification technologies for the treatment of PAD.

## Arterial Haemodynamics

The haemodynamics of arterial blood flow describes the mechanical properties and the interactions between blood flow, vascular wall and perivascular tissue. More specifically, haemodynamics describe the kinematics and dynamics of blood flow and obey the laws that govern the mass and momentum of fluid elements. The first requires the mass to be conserved (continuity), and the second based on Newton’s law of motion, requires the conservation of momentum (Navier–Stokes equations).

Computational fluid dynamics (CFD) is widely used to study the cardiovascular system based on image-based patient specific techniques.^2^ Such CFD applications can be used in diagnosis, therapeutic planning and post-operative monitoring of patients, supporting the clinical decision making process. CFD may also be used in the prediction of medical device performance.^14^

The blood vessels have the ability to respond to haemodynamic stimuli, including the stretch and shear stress resulting from flow and circulatory (transmural) pressure. This ability is mainly mediated by vascular endothelial cells, which are in direct contact with blood flow. Endothelial cells can sense and respond to hemodynamic stresses that are exerted upon them by activating mechano-sensors, signalling pathways, and gene and protein expressions.^12^

Wall shear stress (WSS) in a vessel is the tangential force generated by blood flow. WSS is determined as the product of wall shear rate (WSR) and blood viscosity, where WSR is the spatial gradient of blood velocity normal to the wall.^5^ Two main theories have been reported about the role of WSS in the development of atherosclerosis. The first suggests that atherosclerosis may be developed in regions of high WSS. In 1968, Fry^26^ supported this theory showing that exposure of endothelial cells in shear stress above 379 ± 85 dynes cm^−2^ for an hour resulted in cell swelling, deformation disintegration and finally cell dissolution and erosion. The second theory, which is widely accepted today, denotes that early atheroma coincides with the regions in which arterial WSR is expected to be relatively low.^5^,^8^,^9^ Endothelial cells tend to align in the blood flow direction under physiologic WSS conditions (>15 dynes cm^−2^) inducing endothelial quiescence; however, under low WSS (<4 dynes cm^−2^), which is prevalent at atherosclerosis-prone sites, endothelial cells show no specific alignment.^54^

Despite the systemic nature of its associated risk factors, atherosclerosis is a geometrically focal disease and arises in complex regions such as bifurcations, where disturbed flow fields may be developed. Disturbed flow includes flow recirculation and separation, complex flow patterns, non-uniform WSS distribution containing regions of low and high mean WSS, long particle residence times and stagnation.^13^,^59^ Flow stagnation drastically reduces the arterial WSS and leads the endothelium to down-regulate the release of anti-thrombotic agents and up-regulate the expression of pro-coagulant agents. In general, disturbed flow may cause platelet activation and therefore increase platelet adhesion which is involved in development of NIH and thrombosis.^3^,^56^,^75^ For instance, studies in human vessel branches and bifurcations have shown that lesions are more likely to form along the outer wall of the branch or bifurcation, where WSS is relatively low or oscillating and flow recirculation is observed.^25^,^32^,^48^,^54^

## Secondary Flow Motions in Arteries

During the last few decades it has been well presented that blood flow in medium and large size arteries is spiral. The existence of secondary flow motions in arteries is related to both the physiological function and anatomical structure of the cardiovascular system.

### Heart

The heart is a remarkable structure that displays a helical, transmural and overlapping pattern of myocardial fibres.^43^ The helical structure of myocardium is related with the twisting heart motion during systole and early diastole.^4^,^43^,^73^ A magnetic resonance imaging (MRI) study by Jung *et al*.^40^ showed that during the spiral-compressive heart function the myocardium follows a helical descent motion. The twisting motion during contraction and early dilation generates a spiral blood pattern in the ascending aorta,^42^,^72^ that may provide more efficient cardiac emptying and reduce the myocardium effort.^67^

### Aorta

The existence of secondary flow motions in the ascending aorta was presented in 1987 by Segadal and Matre^64^ with a transluminal Doppler ultrasound probe. Three years later Frazin *et al*.^23^ used colour Doppler ultrasound to show rotational flow in an aortic arch model and human descending thoracic aorta. Their main findings were a clockwise systolic rotation and a counter-clockwise diastolic rotation. On a *post hoc* study, they presented spiral flow in the abdominal aorta beyond the renal arteries.^24^ More recently, spiral flow was confirmed in the ascending and abdominal aortas using vector Doppler.^63^,^72^

The development of phase contrast MRI verified clockwise helical flow in the aortic arch and abdominal aorta of healthy volunteers during systole and showed a second retrograde rotational flow along the inner wall at end systole.^34^,^35^,^42^,^55^

### Peripheral Arteries

**Figure 1:**
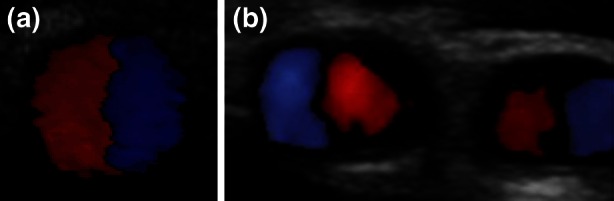
Single spiral flow detected with colour Doppler ultrasound; (a) femoral artery; (b) internal and external carotid arteries.

Ku and Giddens,^47^ and Karino *et al*.^41^ showed the development of secondary flow motions in realistic peripheral arterial models as a result of bifurcations and branching. Complicated vortical structures were associated with flow separation and stagnation. In 1991, Stonebridge and Brophy^68^ detected spiral flow in peripheral arteries of the lower extremities and were the first to conclude that flow in arteries may be spiral in nature. Since that, the presence of spiral flow in right and left iliac and femoral arteries of healthy volunteers has been confirmed by several researchers using colour Doppler and vector Doppler imaging.^31^,^63^,^70^ Spiral flow can be detected using colour Doppler ultrasound with the transducer perpendicular to the vessel and appears as a red–blue split (Fig. 1).^31^ Caro *et al*.^7^ used phase contrast MRI to present rotating asymmetric axial velocity profiles in iliac and carotid arteries. In addition, the existence of rotational patterns distal from the carotid bifurcation have been confirmed with both MRI and ultrasound imaging techniques.^29^,^74^

### Maintenance of Spiral Flow in Arteries

The propagation of spiral flow in the arterial system is supported by its multi-planar, curvy, tapering and branching geometry.^7^,^24^,^70^ Numerical simulations in aortic arch models showed that a curved but planar arch would exhibit a typical Dean flow with two symmetric opposite helical structures, instead of a single spiral flow which is the clinical observation, indicating the importance of aortic torsion.^52^,^53^ Stonebridge and Brophy^68^ detected spiral folds on arterial walls downstream from the aortic bifurcation and postulated that such folds may support the propagation of spiral flow.

### Clinical Correlation of Arterial Spiral Flow

Frazin *et al*.^24^ speculated that the tangential component of the aortic blood flow affects the wall shear forces and endothelial cell alignment and may play an important role in plaque deposition, organ perfusion, dissection formation and flow separation in aortic branches. Numerical studies supported the hypothesis that the aortic spiral flow may enhance oxygen and nutrient flux to the arterial wall.^52^,^58^ Other computational^53^ and *ex vivo*^19^ studies showed that helical flow may suppress the luminal surface concentration polarisation of low-density lipoproteins, which is involved in the localisation of atherogenesis. Zhan *et al*.^82^ presented that spiral flow, in comparison to parabolic flow, decreased the adhesion of platelets in a test tube and concluded that spiral flow may decrease the risk of acute thrombus formation in vascular implants. Their results were supported by Massai *et al*.^56^ who showed an inverse relationship between platelet activation and helical flow in a stenosed carotid model using CFD.

It is believed that the helical velocity of spiral flow is an important stabilising factor of WSS magnitude and dispersion and therefore may have a beneficial effect on the mechanisms of endothelial damage and repair.^7^,^24^,^70^,^72^ Morbiducci *et al*.^57^ presented an inverse relationship between OSI and helical flow. They hypothesised that helical flow may damp WSS temporal gradient in aortocoronary bypass. Chen *et al*.^10^ compared helical and parabolic flow in a stented region and found that helical flow reduced the size of disturbed flow zones, increased the average WSS and lowered the OSI, which could suppress the development of restenosis after stent implantation. Caro *et al*.^6^ demonstrated that a helical blood pattern may protect vessels from NIH and thrombosis because it introduces uniform WSS, increases blood flow mixing, and reduces flow stagnation, separation and instability. Other studies linked the arterial three-dimensionality with reduction of low WSS regions and increase oxygen flux protecting the endothelium.^16^,^17^ Spiral flow was also compared with parabolic flow in stenosed straight tube models. The former flow pattern was associated with a significant reduction of turbulent kinetic energy and capability to preserve a coherent pattern downstream of the stenosis, suggesting flow stabilisation even in stenosed vessels.^61^,^69^

In a clinical study, Houston *et al*.^35^ linked the reduction of systolic spiral flow in the aortic arch with carotid atheromatous disease. In a further clinical study, they concluded that the lack of aortic spiral flow in patients with renal arterial stenosis is related with significant short-term renal function deterioration.^34^

It has been noted that spiral flow may also have a detrimental role in the cardiovascular system.^28^,^68^ For instance, the spiral folds detected on arterial walls^68^ can be associated with helical distribution of low WSS.^7^ The potential link between shear forces induced by rotational flow and plaque deposition was also noted by Frazin *et al*.^23^ Paul and Larman^61^ applied spiral and parabolic flow in a stenosed numerical model and found increased OSI for spiral flow. Other studies demonstrated that spiral flow can increase pressure drop and showed a proportional relationship between pressure drop and helicity.^10^,^15^,^44^

In order to prove the exact correlation between spiral flow and atheromatous plagues, there is still a need for long-term randomised clinical data where the relationship between flow patterns and development of arterial disease will be characterised.^59^,^67^

## Introducting Flow Optimisation to Stent Design

Stent design plays an important role in development of in-stent restenosis and downstream progression of PAD after implantation. Stent struts denude most of the endothelium in contact and re-endothelialisation is required. The endothelial damage is increased in the case of a balloon expanded stent, because of the extensive contact between foreign materials and the arterial wall.^30^,^77^ Arterial stress from stent deployment does not only injure the endothelium but also stretches the media and adventitia, which can disrupt the stent incorporation process.^22^ If a stent is oversized medial injury is possible, due to high stress against the wall, initiating early thrombosis and acute inflammation.^21^

Local haemodynamics is another mechanism associated with both in-stent restenosis and distal disease progression, because a stent alters the haemodynamics in both the stented region and downstream from it. The presence of a stent in infrainguinal arteries induces stagnation, recirculation zones, areas of low, high or oscillatory WSS and prolonged particle residence time, creating an unstable environment and disturbing the physiologic spiral flow.^11^,^39^,^59^,^77^ Wentzel *et al*.^78^ applied CFD in stented coronary arteries and showed an inverse relationship between in-stent neointimal thickness formation and WSS magnitude.

Haemodynamics are affected by both stent oversizing and undersizing. Stent oversizing increases the lumen diameter and consequently reduces the blood flow velocity and WSS in the stented region. It also creates a step-up phenomenon at the edges of the device developing recirculation zones. On the other side stent undersizing may leave tiny gaps between the struts and the arterial wall, which introduces flow resistance and increases WSS gradient and OSI in comparison to correct-sized cases.^11^,^59^ The patency of a stent is also dependent on the pre-operative arterial geometry. For example, stent revascularisation of a curvy artery may cause arterial straightening. This would result in arterial kinking at the edges of the stent, inducing large spatial and temporal variations of WSS and increased arterial trauma, supporting the development of NIH.^50^,^79^

Stent design characteristics such as stent strut pattern, struts spacing, thickness and roughness affect both arterial trauma and local haemodynamics. LaDisa *et al*.^51^ found that a reduction of struts number, thickness and width leaves a higher percentage of arterial wall intact and elevates WSS. This reduction would also decrease the extent of arterial trauma. Jimenez and Davies^39^ proposed a streamlined stent strut design in order to reduce recirculation zones and therefore prevent thrombosis and support re-endothelialisation.

Another important characteristic of infrainguinal stents is their material properties and specifically their ability to withstand movements and flexions of the arteries as a result of everyday activities of a patient. The incidence of infrainguinal stent fracture ranges between 2 and 65%. Stent fracture may cause further haemodynamic disturbances and a new wall injury leading to restenosis.^18^,^22^

## Flow Modification Stents

Research on physiologic spiral flow has motivated two manufacturers to develop peripheral arterial stents capable of generating spiral flow. These devices aim to re-introduce spiral flow in order to reduce NIH and progression of atherosclerosis, and therefore provide improved stent and vessel patency rates for patients with PAD.

### Spiral Flow Stent

**Figure 2:**
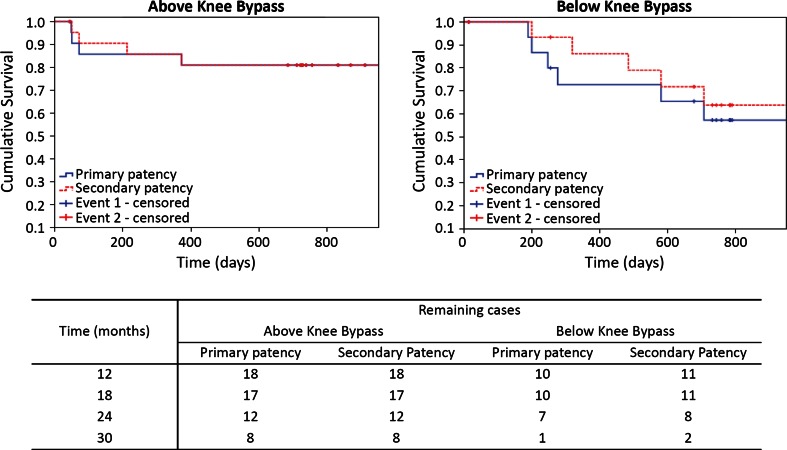
Primary and secondary patency rates of above- and below-the-knee bypasses of spiral graft for PAD.^71^

Results of research that spiral flow may create a stabilised, coherent and protective blood flow field, led Vascular Flow Technologies Ltd. (VFT, Dundee, United Kingdom), to develop Spiral Flow™ vascular implants. Spiral flow technology was originally applied in prosthetic grafts for the treatment of PAD and for arteriovenous dialysis conduits. These devices are supported by an internal spiral inducer ridge in their distal end in order to generate spiral blood flow and have received CE mark and FDA (Food and Drug Administration) approval. In 2012, Stonebridge *et al*.^71^ presented a nonrandomised multicentre clinical study on 39 patients who received spiral grafts for the treatment of PAD. At 30 months the primary and secondary patency rates were 81% for above-the-knee bypasses and 57 and 64% respectively for below-the-knee bypasses (Fig. 2). At the time of the publication these patency rates were better or similar to other relevant studies. Encouraging data were also presented for the arteriovenous spiral graft in an animal^38^ and two clinical studies.^20^,^37^ In the first clinical study, Inston *et al*.^37^ verified the development of spiral flow in the distal anastomosis of a spiral graft and showed low incidence of outflow stenosis and 75% primary patency at a mean time point of 188 days.^37^ In the second clinical study, El-Sayed^20^ presented assisted primary and secondary patency rates of 70 and 82% respectively. These patency rates were significantly better than the historic data on conventional grafts in their institution.^20^ The authors in both studies hypothesised that these results were associated with the coherent single spiral flow in the venous graft anastomosis.

**Figure 3:**
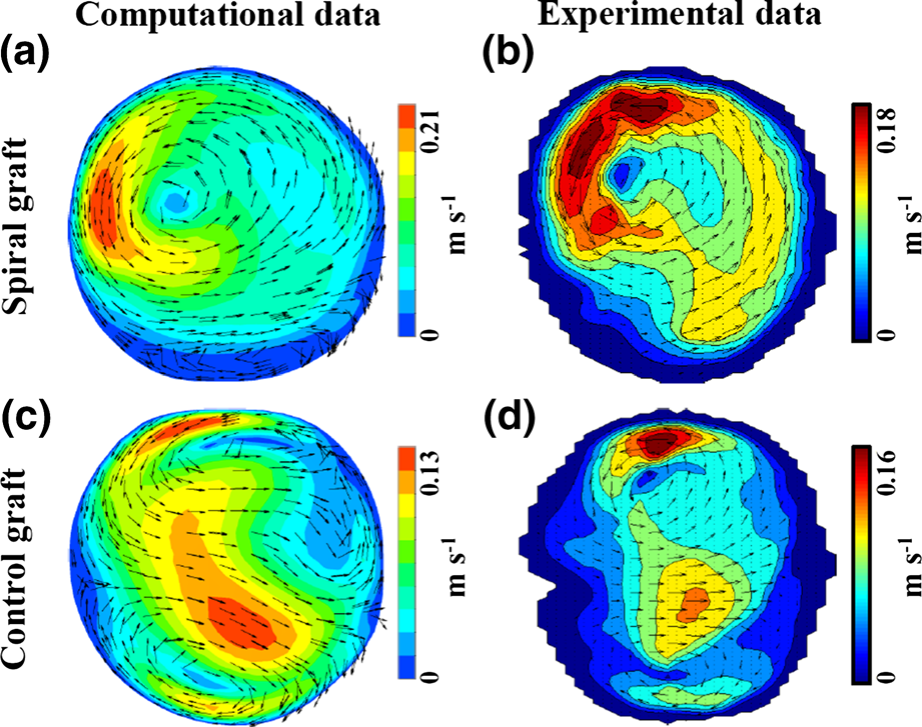
Cross section velocity maps in the outflow anastomosis of a spiral and a conventional graft for PAD, tested under the same geometry and flow conditions in flow phantoms. Velocity maps were estimated with CFD and detected with vector Doppler ultrasound. (a, b) A dominant single spiral with a second smaller spiral below (mainly seen in the computational map). (c, d) Double spiral.^44^

In addition, the spiral grafts were compared with conventional grafts in idealised flow phantom setups, using experimental and computational approaches.^44^–^46^ A dominant single spiral was found in the distal anastomosis of the spiral graft for PAD as well as a second small flow recirculation which was dissipated a few centimetres downstream (Figs. 3a and 3b). On the other hand a double spiral (Dean vortex) was found in the distal anastomosis of the conventional graft (Figs. 3c and 3d). The secondary flow phenomena were stronger for the spiral device as was seen by the greater in-plane velocities (Fig. 3) and was verified comparing the helicity in the outflow of the tested grafts.^44^ These results showed that a spiral graft can reduce points of flow stagnation breaking the problematic symmetry of a Dean vortex profile which is observed in conventional grafts,^60^,^65^ and introduce a stabilised flow field.

**Figure 4:**
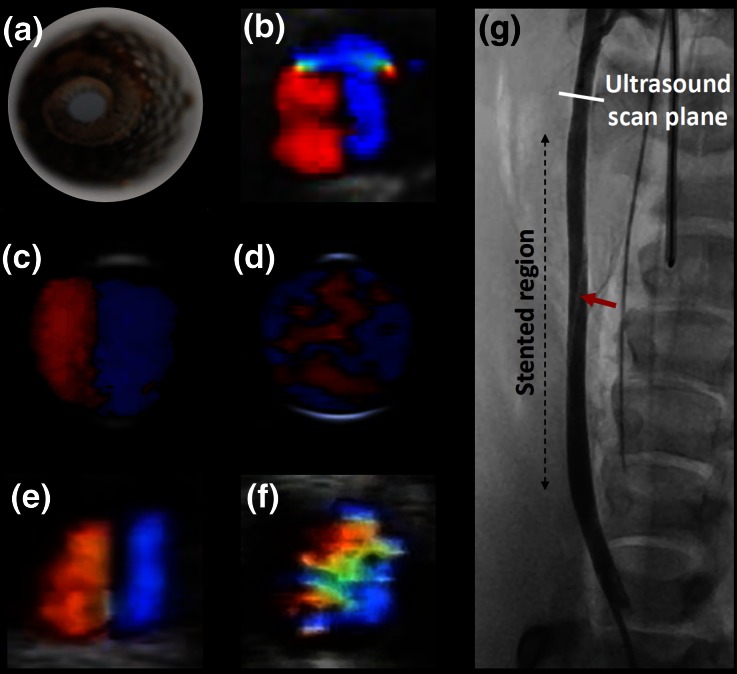
(a) Spiral flow self-expanding stent prototype inserted in a testing tube. (b) Single spiral in the outflow of a spiral stent implanted in a cadaveric model; steady flow was used. (c–d) Single spiral and non-spiral flow in the outflow of a spiral and a control stent respectively, positioned in a flow phantom; steady flow was used. (e–f) Single spiral and non-spiral flow in the outflow of a spiral and a control stent respectively, implanted in porcine models; images were obtained at peak systole. (g) Contrast angiogram of spiral stent implanted in the right carotid artery of a pig; the red arrow indicates a visible part of the spiral inducer and the cross-section white line the ultrasound scan plane distal from the outflow of the stent.

The re-introduction of naturally occurring spiral arterial flow in vascular grafts with improved clinical results and long-term safety at 5 years post implantation, has led to development of an arterial spiral inducing stent for the treatment of PAD. This device has a conventional cylindrical outside geometry, where a part of the stent struts form a spiral internal ridge, and it is covered with polytetrafluoroethylene (Fig. 4a). This spiral ridge is configured to drive the blood in a spiral flow pattern which is believed to protect the artery from restenosis and PAD progression.

Such prototypes were housed in stent delivery systems and implanted in ultrasound flow phantoms, pigs (with approval by the Animal Experimentation Committee, Faculty of Medicine, Utrecht University, the Netherlands and in accordance with the Netherlands guidelines on animal care) and cadaveric models (ethical approval was obtained from the University of Dundee Thiel Advisory Group and the study was complied with the Anatomy Act (Scotland) 2006). In the first two cases spiral stents were compared with control stents of same dimensions and struts design. Steady flow was applied in the flow phantoms and cadaveric models. Spiral flow was found in the outflow of all spiral stents and non-spiral flow pattern in the outflow of the control stents as seen in Figs. 4b–4f. Figure 4g shows a contrast-enhanced angiogram of a spiral stent implanted in a porcine model and the location of the ultrasound scan plane downstream from the outflow of the stent. The current evaluation of this device includes CFD simulations and longitudinal histological studies in stenosed and non-stenosed porcine models, as well as optimisation of the stent struts design for increased durability and flexibility.

**Figure 5:**
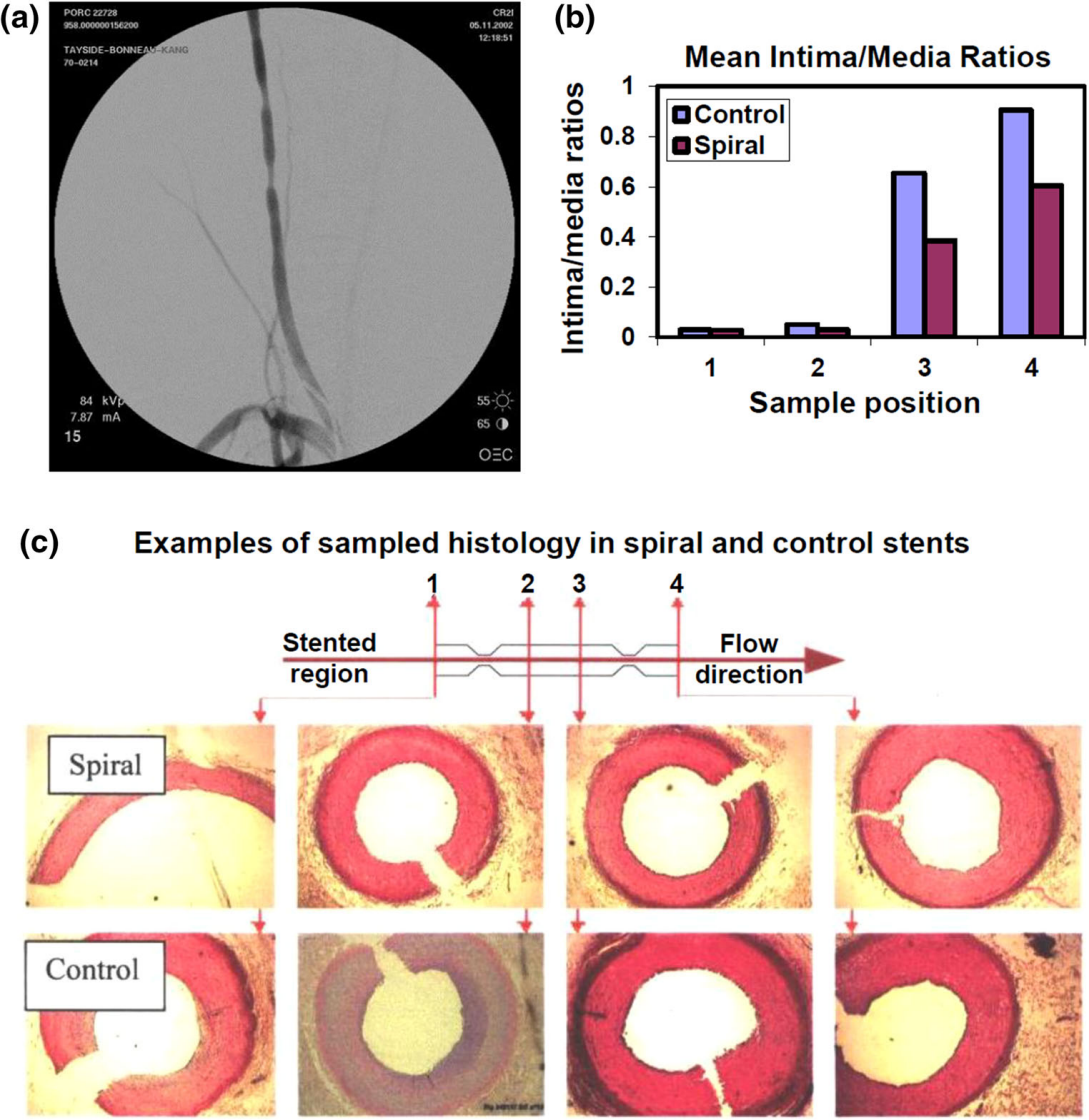
Preclinical porcine study of carotid balloon expandable spiral stent compared to control stent. (a) Digital subtraction angiogram post stent implantation with downstream cuffs. (b) Intima media ratio comparison of spiral versus control stent. (c) Sample histology in four locations downstream from spiral and control stents shows increased neo-intimal hyperplasia in control stent.^33^

In a previous similar spiral stent approach Houston *et al*.^33^ incorporated a collapsible spiral induction form made from medical grade polyurethane in 16 commercially available balloon expandable stainless steel stents. The spiral stents were remounted in delivery systems and resterilised for implantation. These spiral stents were compared with 16 control stents in carotid porcine models. More specifically, a spiral and a control stent were implanted in the carotid arteries of 16 pigs. Two surgical cuffs were placed around each artery distal from the stented region as shown in Figs. 5a and 5c, to analyse the progression of downstream disease development for the spiral and control stents. These cuffs created a minimum 50% stenosis (Fig. 5a). Cross-section colour Doppler imaging was applied distally from the stents to detect the flow pattern profile. Histological analysis was carried out in the locations shown in the diagram of Fig. 5c. Spiral flow was found in the outflow of spiral stents whereas disturbed flow was found in the outflow of control devices. Histological analysis 45 days post-operatively indicated significantly reduced intima/media ratios and reduced downstream neo-intimal thickening in spiral versus control stents as shown in Figs. 5b and 5c respectively.^33^

### BioMimics 3D Vascular Biomimetric Stent

**Figure 6:**
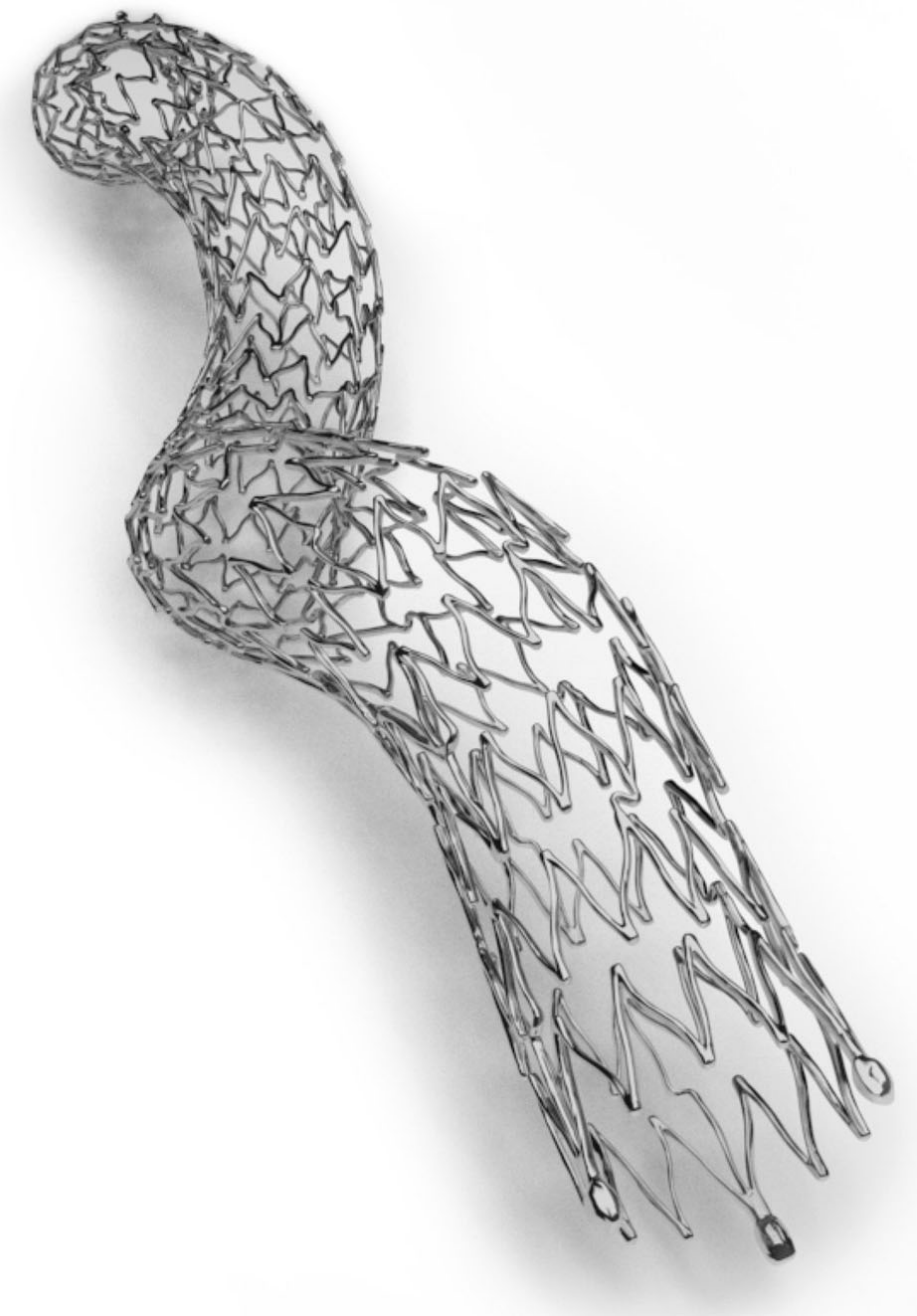
BioMimics 3D helical stent (reproduced with permission of Veryan Medical Ltd).^76^

The BioMimics 3D™ vascular biomimetric stent has been developed by Veryan Medical Ltd (West Sussex, United Kingdom) for the treatment of superficial femoral and proximal popliteal arteries. This device has a 3D helical shape (Fig. 6) in order to maintain and propagate the physiologic helical flow. According to Veryan Medical the helical shape of the BioMimics 3D stent decreases fracture incidents from femoropopliteal arterial shortening during knee bending. It is claimed that the 3D helical geometry prevents the stent from kinking and distributes the compressive strain across the entire stent, in comparison to straight stents which tend to kink creating high localised strain regions. The BioMimics 3D stent has a tapered radial stiffness at its ends, which aims to minimise the possibility of the step-up phenomenon, protecting from flow recirculation, stagnation and low WSS.^76^

A preliminary *in vivo* study of the BioMimics 3D stent was based on porcine models (*n* = 10). Each animal was implanted with a BioMimics 3D stent in one carotid artery and a conventional stent in the other one. The ability of the BioMimics 3D stent to create helical flow was tested with colour Doppler imaging. Histological cross-section tests at 1 month post-implantation demonstrated a 45% reduction in NIH for the helical device.^66^ The device received CE mark in 2012 and positive clinical data were recently presented from a 2-year, multicentre, randomised study in 76 patients (Mimics study).^81^ 50 of them received a BioMimics 3D stent and the rest a control stent. The primary patency at 2-years was 72% for the helical stent and 55% for the control, showing a significant difference by log-rank test. No increase was detected in the clinically driven target lesion revascularisation rate for the BioMimics 3D stent between 12 and 24 months in comparison to a threefold increase for the control stent. The helical stent showed significantly greater curvature in comparison to the straight stent and a correlation between primary patency and stent curvature was hypothesised. No fracture was reported for the BioMimics 3D stent.^81^

The BioMimics 3D has been developed based on research by Caro who hypothesised the atheroprotective effect of high WSS and showed that this is supported by blood helical flow and arterial non-planarity.^5^,^7^–^9^ Helical flow technology was initially applied in a small amplitude helical graft (SwirlGraft™) for arteriovenous applications. This non-planar graft was designed to introduce a single recirculation in its distal end-to-side anastomosis, instead of a symmetric Dean vortex profile which is observed in the outflow of conventional grafts.^60^,^65^ The SwirlGraft showed reduced thrombosis and NIH in a comparison with conventional grafts in porcine models. The authors used CFD data to link the superior *in vivo* results of the SwirlGraft device with relatively uniform WSS, increased in-plane mixing and inhibition of flow separation and stagnation.^6^ In a following non-randomised 6-month-long clinical study by Huijbregts *et al*.,^36^ the helical graft was associated with promising primary (58%), assisted primary (84%) and secondary (100%) patency rates. At the time of publication these patency rates were better or similar to other relevant studies. However, thrombosis incidences exceeded the clinical practice guidelines for vascular access at that time. Angiographic examination suggested that there was a reduction of helical geometry upon implantation and it was hypothesised that this could be related to the increased thrombosis incidences.

## Conclusions

The key role of haemodynamics in arterial wall function is well accepted. The importance of a better understanding of physiologic blood flow and its relevance to optimised arterial stents in PAD is clear. Current research indicates that re-introducing spiral arterial blood flow after the treatment of PAD, has theoretical benefits with the potential to reduce NIH, thrombosis and disease progression. Spiral vascular grafts show encouraging long-term clinical results while initial preclinical and clinical results of flow modification stents are promising. Such stent designs may stabilise the arterial haemodynamics and thereby may contribute to the improvement of clinical outcomes for endovascular treatment of PAD.
